# The biogeography of the caribou lungworm, *Varestrongylus eleguneniensis* (Nematoda: Protostrongylidae) across northern North America

**DOI:** 10.1016/j.ijppaw.2020.01.001

**Published:** 2020-01-08

**Authors:** Guilherme G. Verocai, Eric P. Hoberg, Manon Simard, Kimberlee B. Beckmen, Marco Musiani, Sam Wasser, Christine Cuyler, Micheline Manseau, Umer N. Chaudhry, Cyntia K. Kashivakura, John S. Gilleard, Susan J. Kutz

**Affiliations:** a Department of Ecosystem and Public Health, Faculty of Veterinary Medicine, University of Calgary, 3330 Hospital Drive NW, Calgary, Alberta, T2N 4N1, Canada; b Department of Veterinary Pathobiology, College of Veterinary Medicine & Biomedical Sciences, Texas A&M University, TAMU, College Station, TX, 77843, USA; c Museum of Southwestern Biology, Department of Biology, University of New Mexico, Albuquerque, NM, 87108, USA; d Makivik Corporation, Kuujjuaq, QC, Canada; e Division of Wildlife Conservation, Alaska Department of Fish and Game, 1300 College Road, Fairbanks, AK, USA; f Department of Biological Sciences, Faculty of Science, University of Calgary, AB, Canada; g Center for Conservation Biology, University of Washington, Seattle, WA, USA; h Greenland Institute of Natural Resources, Department of Mammals & Birds, DK-3900, Nuuk, Greenland; i Natural Resources Institute, University of Manitoba, Winnipeg, Manitoba, Canada, R3T 2M6; j Department of Comparative Biology and Experimental Medicine, Faculty of Veterinary Medicine, University of Calgary, 3330 Hospital Drive NW, Calgary, Alberta, T2N 4N1, Canada

**Keywords:** Arctic parasitology, Climate change, Geographic distribution, Metastrongyloidea, Nearctic, *Rangifer*

## Abstract

*Varestrongylus eleguneniensis* (Nematoda; Protostrongylidae) is a recently described species of lungworm that infects caribou (*Rangifer tarandus*), muskoxen (*Ovibos moschatus*) and moose (*Alces americanus*) across northern North America. Herein we explore the geographic distribution of *V. eleguneniensis* through geographically extensive sampling and discuss the biogeography of this multi-host parasite. We analyzed fecal samples of three caribou subspecies (n = 1485), two muskox subspecies (n = 159), and two moose subspecies (n = 264) from across northern North America. Protostrongylid dorsal-spined larvae (DSL) were found in 23.8%, 73.6%, and 4.2% of these ungulates, respectively. A portion of recovered DSL were identified by genetic analyses of the ITS-2 region of the nuclear rDNA or the cytochrome oxidase c subunit I (COI) region of the mtDNA. We found *V. eleguneniensis* widely distributed among caribou and muskox populations across most of their geographic prange in North America but it was rare in moose. *Parelaphostrongylus andersoni* was present in caribou and moose and we provide new geographic records for this species. This study provides a substantial expansion of the knowledge defining the current distribution and biogeography of protostrongylid nematodes in northern ungulates. Insights about the host and geographic range of *V. eleguneniensis* can serve as a geographically extensive baseline for monitoring current distribution and in anticipating future biogeographic scenarios under a regime of accelerating climate and anthropogenic perturbation.

## Highlights

• *Varestrongylus eleguneniensis* is a lungworm whose primary host is the caribou.
• The muscleworm, *Parelaphostrongylus andersoni*, co-infects caribou across its range.
• We expand the knowledge on distribution of the caribou lungworm and the muscleworm.
• Muskoxen sympatric with caribou are infected with the caribou lungworm.
• We discuss the biogeography of *V. eleguneniensis* and *Rangifer* across North America.

## Graphical abstract

**Figure:**
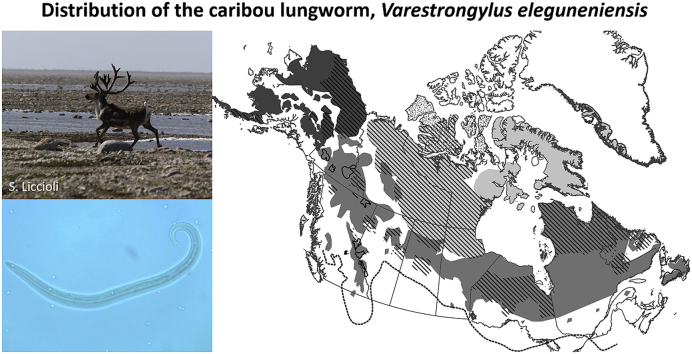

## 1 Introduction

The biogeography of a parasite species is directly influenced by the distribution of its hosts (definitive and intermediate) species and the nature of environmental conditions which determine development and opportunity for transmission. Thus, where particular parasite species occur is the product of complex historical, evolutionary and ecological processes that have occurred through space and time. Climatic fluctuations, for example, are among the primary drivers that have shaped biodiversity and have served as determinants of current host-parasite associations (Hoberg, 2010; Hoberg and Brooks, 2008, 2013; 2015; Hoberg et al., 2012). The dynamic processes driving host-parasite associations and biogeography are not only part of history, but continue to act across the biosphere today (Altizer et al., 2013; Hoberg et al., 2012, 2017; Kutz et al., 2009, 2012; Parmesan and Yohe, 2003). Processes that once unfolded on millennial scales, or thousands of years, are now occurring at a much faster pace in a world influenced by direct and indirect anthropogenic drivers, including climatic perturbations, landscape modifications and animal movement, impacting both host and parasite biogeography (Hoberg and Brooks, 2008, 2010, 2015; Hoberg et al., 2017). An understanding of processes that formed host-parasite associations and biogeography in evolutionary and ecological time provides a pathway to explore the implications of accelerating environmental perturbations today (Hoberg and Brooks, 2015; Hoberg et al., 2012; Kutz et al., 2014).

Caribou, *Rangifer tarandus* (L.), is an iconic species in North America, with a complex of subspecies and populations ranging from the boreal forests of Canada and USA to the islands of the High Arctic, and Greenland. Across North America, numerous caribou populations of different subspecies and ecotypes are facing declines, and their recovery and sustainability are uncertain (Gustine et al., 2014; Hervieux et al., 2013; Vors and Boyce, 2009). This raises serious concerns about species conservation and long-term persistence, with various populations assessed as threatened and endangered (COSEWIC, 2004, 2011; 2016; Festa-Bianchet et al., 2011). Despite the ecological importance of caribou, parasite biodiversity in these keystone ungulates has been relatively understudied (reviewed in Kutz et al., 2012).

Among parasites in caribou, nematodes of the Family Protostrongylidae Leiper, 1926 are of concern as potential pathogens that can influence morbidity, mortality and host demographics (Kutz et al., 2012; Lankester, 2001). Depending on the species, adult protostrongylid nematodes inhabit the respiratory tract, skeletal muscles or the central nervous system of their definitive hosts, causing parasitic pneumonia and/or debilitating muscular or neurological disease (Anderson, 2000; Kutz et al., 2012; Lankester, 2001). The recent discovery of a new, wide-spread, protostrongylid lungworm, *Varestrongylus eleguneniensis* Verocai, Kutz, Simard & Hoberg, 2014b, has reinforced the need for a comprehensive assessment of protostrongylid biodiversity in caribou as a basis for understanding the complexities of this host-parasite assemblage (Kutz et al., 2007, 2012; Verocai et al., 2014b). In addition to caribou, *V. eleguneniensis* also infects muskoxen (*Ovibos moschatus* Zimmermann) and, incidentally, moose (*Alces americanus* (L*.*) (Kutz et al., 2007, 2012, 2013; Verocai et al., 2014b). It has been reported in these species at various arctic and temperate locations across North America (Kutz et al., 2007; Verocai et al., 2014b, Kafle et al., 2017a, Kafle et al., 2017) and recently has also been observed expanding on a northward trajectory crossing the Northwest Passage, and establishing on Victoria Island in the Arctic Archipelago, Canada (Kutz et a. 2013a; Kafle et al., 2017a, Kafle et al., 2017).

Another protostrongylid nematode which sympatrically infects caribou along with *V. eleguneniensis* is the muscle worm, *Parelaphostrongylus andersoni* Prestwood, 1972. In northern latitudes of North America (Kutz et al., 2007; Lankester and Hauta, 1989; Verocai et al., 2013), *P. andersoni* infects caribou and moose, but it is also reported in white-tailed deer, *Odocoileus virginianus* Zimmermann, over an extensive, but apparently discontinuous, range across North America (Asmundsson et al., 2008; Lankester, 2001; Prestwood, 1972).

These early advances of knowledge *V. eleguneniensis* lead us to further investigate its biogeography at a finer scale focusing on caribou from Alaska to Greenland, but also sympatric muskox and moose populations across boreal forest and tundra ecosystems. We discuss the complexities of this dynamic system in light of historical processes and current trends in host populations, ongoing range shifts, and potential future scenarios. In addition, we provide substantial information on the biogeography of, *P. andersoni* in caribou and moose, as well as findings on other protostrongylid species.

## 2 Material and methods

### 2.1 Sample acquisition

Fecal samples were collected in the field from high latitude ungulates: (1) three subspecies of caribou, [Grant's, *Rangifer tarandus granti* (Allen, 1902); barren-ground, *Rangifer tarandus groenlandicus* (Borowski, 1780), and woodland caribou, *Rangifer tarandus caribou* (Gmelin, 1788)] (Table 1, S1, S2, S3); (2) the two subspecies of muskoxen from across Canada, Alaska and Greenland, namely *Ovibos moschatus moschatus* (Zimmermann, 1780), and *Ovibos moschatus wardi* Lydekker, 1900 (Table 2); and (3) two subspecies of moose, *Alces americanus andersoni* Peterson, 1952, and *Alces americanus gigas* Miller, 1889 (Table 3). In summary, a total of 1485 caribou samples collected between March 1998 and March 2012; 159 muskoxen samples collected between May 2007 and April 2011; and 264 moose samples collected between March 2019 and March 2012 were included in this study (Table 1, S1, S2, S3). Specimens were acquired from localities in western Canada and Alaska through a wide network of collaborators with research, government, and co-management institutions at regional, provincial, territorial and state levels. Acquisition and use of samples were covered under Permit # AC13-0121 from the Animal Care Committee of the Faculty of Veterinary Medicine of the University of Calgary. Because of the controversial taxonomic classification of caribou, we followed the currently accepted subspecies designations, but also included the ‘ecotypes’ for each herd according to Festa-Bianchet et al. (2011). Samples were collected opportunistically when animals where handled for other projects (e.g., capture, collaring or translocation) or harvested for scientific research or by subsistence hunters. Where major geographic gaps existed, increased efforts were made to acquire samples through collaborating wildlife biologists and veterinarians with government and researchers.

**Table 1:** Total caribou (*Rangifer tarandus* sspp.) fecal samples included in the study: information on subspecies and origin, and Baermann results (prevalence of dorsal-spined larvae; DSL). Molecular identification of DSL was based on sequences of the ITS-2 region of the nuclear ribosomal DNA. See Tables S1, S2, and S3 for results of individual herds of Grant's, barren-ground and woodland caribou, respectively.

Caribou subspecies	N	DSL (%)	*V. ele.* (DSL/host)	*P. and.* (DSL/host)
***R. t. granti***	203	54 (26.6)	13; 8	77; 32
***R. t. groenlandicus***	375	96 (25.1)	10; 8	5; 3
***R. t. caribou***	907	212^a^ (23.4)	69; 36	193; 72
**Total**	**1485**	**354 (23.8%)**	**91; 51**	**275; 107**

a The overall frequency of DSL includes caribou infected with *Elaphostrongylus rangiferi* (Newfoundland caribou herds) and undetermined *Parelaphostrongylus* species (some British Columbia woodland caribou herds). *V. ele*. = *Varestrongylus eleguneniensis*, *P. and*. = *Parelaphostrongylus andersoni*.

**Table 2:** Muskox (*Ovibos moschatus* sspp.) fecal samples included in the study: information on subspecies and origin, and Baermann results (prevalence of dorsal-spined larvae; DSL). Molecular identification of *Varestrongylus eleguneniensis* larvae were determined based on sequences of the mitochondrial **cytochrome *c* oxidase subunit 1** (COI).

Subspecies/Range	Region/Game Unit	Month, Year	N	DSL (%)	*V. ele.* (DSL; host)	Comments	
***Ovibos m. wardi***							
**Endemic**							
Greenland	West Greenland	April 2009	6	0 (0)	–	–	
Nunavut	Ellesmere Isl.	July–Aug 2010	4	0 (0)	–	–	
**Introduced**							
Alaska	GMU 22C	April 2010	1	0 (0)	–	–	
	GMU 26B	June 2010	1	0 (0)	–	–	
	GMU 23	March 2011	6	6 (100)	6; 6	All also + for *P. stilesi*	
	GMU 22E	March 2011	4	4 (100)	4; 4		
	GMU 26B	March 2011	5	0 (0)	0	All adults	
	GMU 22E	March 2011	3	2 (66.7)	2	Adult females	
	GMU 23	March 2011	2	0 (0)	0	–	
	GMU unknown	March 2011	1	0 (0)	0	–	
	GMU 22D	April 2011	13	13 (100)	NA	All adults	
	GMU 26B	April 2011	1	0 (0)	–	+ for *P. stilesi*	
Quebec^a^	Nunavik	Winter 2008	15	13 (86.7)	+	Previous work	
		April 2009	7	5 (71)	+	Previous work	
		Dec 2009	1	1 (100)	+	(Verocai et al., 2014a, Verocai et al., 2014b)	
		Jan 2010^a^	2	2 (100)	+	(Verocai et al., 2014a, Verocai et al., 2014b)	
		March 2010	2	2 (100)	+	(Verocai et al., 2014a, Verocai et al., 2014b)	
		April 2011^a^	20	14 (70)	9; 9	All adults	
**Total *O. m. wardi***			**94**	**64 (68)**	**19; 19**		
***Ovibos m. moschatus***							
**Endemic**^b^							
Nunavut	Kugluktuk	May–Aug 2007	57	47 (82.5)	NA^c^	*U. pallikuukensis* range	
Northwest Territories	Sahtu	Feb 2011	8	8 (100)	NA^c^	*U. pallikuukensis* range	
**Total *O. m. moschatus***			**65**	**55 (84.6)**	**NA**^c^		
**TOTAL *O. moschatus***			**159**	**117 (73.6)**	**19; 19**		

+ = Indicates that the identity of larvae from these animals was confirmed by sequencing of the ITS-2 region instead of COI. Material from these collections (adult and larval nematodes) were used for the taxonomic description of the species (Verocai et al., 2014a, Verocai et al., 2014b, Chapter 3), and therefore consist in the type series of *V. eleguneniensis*.

a Additional fecal samples of Nunavik muskox were collected by helicopter on the tundra in April 2010 and April 2011. All herds examined were positive for *V. eleguneniensis*. Material of these tundra collections along with material from the January 2010 collection and of the April 2011 experimental hunt were used for experimental infections of reindeer and muskoxen for elucidating the life cycle of the species (Kafle et al., 2017b).

b Additional hundreds of muskoxen fecal samples from Victoria Island, shared by Nunavut and the Northwest Territories were also analyzed. Larvae of both V. eleguneniensis and U. pallikuukensis were isolated and sequenced at the ITS-2 region, including a case of co-infection by the two protostrongylid species. Results are published in Kutz et al. (2013).

c DSL not sequenced because animals from these areas were already known to be infected by V. eleguneniensis as per Kutz et al. (2007). In addition, these populations are largely sympatric with infected barren-ground caribou herds (Kutz et al., 2007, 2013; Present Study). GMU = Game Management Unit., V. ele. = Varestrongylus eleguneniensis.

**Table 3:** Moose (*Alces americanus* sspp.) fecal samples included in the study: information on subspecies and origin, and Baermann results (prevalence of dorsal-spined larvae; DSL). Molecular identification of DSL was based on sequences of the ITS-2 region of the nuclear ribosomal DNA.

Subspecies/Range	Region/Game Management Unit	Month, Year	N	DSL (%)	***V. ele.* (DSL; host)**	***P. and.* (DSL; host)**	Comments
***Alces a. gigas***							
**Alaska**	GMU 22C	April 2010	30	2 (6.7)	–	–	Not determined. All 10mo. males
	GMU 26A	April 2010	20	0	–	–	All adult females
	GMU 20A	April 2010	2	0	–	–	No info
	GMU 20D	April 2010	2	0	–	–	No info
	MRC	Aug 2010	2	0	–	–	All calves
	GMU 16A/14B	Aug 2010	1	0	–	–	Female calf
	GMU 20B	Oct 2010	1	0	–	–	Male calf
	GMU 14	Jan–Feb 2011	3	0	–	–	All calves
	GMU 20C	March 2011	18	0	–	–	All 10mo calves
	GMU 24B	April 2011	34	1 (2.9)	–	–	Not determined. All adult females
	GMU 15	May 2011	1	0	–	–	Adult female
	GMU 20A	Oct 2011	36	3 (8.3)	–	3; 2	All adult females
	GMU 11	Oct 2011	9	2 (22.2)	1; 1	5; 1	All Adults
	GMU 12	Oct 2011	6	1 (16.7)	–	2; 1	Adult female
	GMU 9E	Oct 2011	1	0	–	–	Adult female
	GMU 20A	March 2012	34	0	–	–	All calves
	GMU 20D	March 2012	32	0	–	–	All calves
	GMU 20C	March 2012	13	0	–	–	All adult females

**Total *A. a. gigas***			**245**	**9 (3.7)**	**1; 1**	**10; 4**	
***Alces a. andersoni***							
**Northwest Territories**	Sahtu	March 2009	8	0	–	–	
	Sahtu	2010/2011	9	1 (11)	–	1; 1	Adult
**Alberta**	Peace River	2011	2	1 (50)	–	1; 1	Adult Male^a^
**Total *A. a. andersoni***			**19**	**2 (10.5)**		**2; 2**	

**TOTAL** ***A. americanus* sspp.**			**264**	**11 (4.2)**	**1; 1**	**12; 6**	

V. ele. = *Varestrongylus eleguneniensis*, P. and. = *Parelaphostrongylus andersoni*, GMU = Game Management Unit.

a The other animal, a yearling male was infected by *Orthostrongylus macrotis*.

### 2.2 Fecal analyses

Fecal samples of all three wild ungulate species were evaluated for the presence of protostrongylid dorsal-spined larvae (DSL) using the beaker Baermann technique (Forrester and Lankester, 1997; Verocai et al., 2013). For caribou and muskoxen, approximately 5 g feces were used for each Baermann, and for moose, because a single pellet often weighed up to 5 g, we used from 5 to 10 g, according to availability. Larvae from each positive host were quantified and stored in water and frozen at −20 °C.

### 2.3 Molecular identification

The goal was to determine presence of *V. eleguneniensis* in a host herd, subspecies or a given location. The general study design for the molecular identification of DSL of infected animals was to initially subsample up to 5 DSL from 5 positive animals of each population, however, there were some exceptions for that effort. For instance, once an animal was confirmed positive for *V. eleguneniensis*, no other animal from that herd was sampled, unless samples of several animals were processed simultaneously. For some populations, only a few larvae were recovered and/or fewer animals were DSL-positive; specifically, for moose and some caribou populations.

DSL isolated from feces were initially collected and placed individually in 0.2 mL tubes containing 5 μL of deionized H_2_O, and subsequently frozen at −80 °C for approximately 15 min. Genomic DNA (gDNA) of individual DSL was extracted in tubes containing 25 μL of lysis buffer (50 mM KCl, 10 mM Tris (pH 8.3), 2.5 mM MgCl2, 0.45% Nonidet P-40, 0.45%

Tween 20, 0.01% (w/v) gelatine and proteinase K at 200 μg/ml). All lysates were kept at −80 °C for a minimum of 10 min before incubation at 60 °C for 98 min, followed by 20 min at 94 °C to denature the proteinase K and stored at −80 °C. 1 ml of 1:5 dilution of lysate DNase, RNase free deionized H_2_O was used as a PCR template. Dilutions of several aliquots of lysate buffer, made in parallel, were used as negative controls.

PCR of individual DSL was performed targeting the ITS-2 region of the nuclear ribosomal DNA using primers NC1 (5′-ACGTCTGGTTCAGGGTTGTT-3ʹ) and NC2 (5ʹ-TTAGTTTCTTTTCCTCCGCT-3ʹ) according to Verocai et al. (2013). Each 20 μL reaction consisted in 10.2 μL of sterile ddH_2_O, 4 μL of 5x PCR buffer + 2 mM MgCl_2_, 0.4 μL of 200uM dNTPs, 2 μL (0.2uM) of each primer, 0.2 μL of 1U *Taq* Phusion HF DNA polymerase, and 1 μL of diluted DNA lysate was added. The amplification conditions used were an initial 2min denaturation at 98 °C, followed by 35 cycles of 98 °C for 10s, 52.5 °C for 30s, and 72 °C for 30s. A final extension of 72 °C for 5min was followed by cooling to 4 °C.

DSL isolated from some muskox populations had the cytochrome oxidase subunit 1 (COI) region of the mitochondrial DNA amplified. PCR of individual DSL was performed using primers PtCOI–F (5′-GGTTGGAGAGTTCTAATCATAAAGA-3ʹ) and VeCOI-R (5ʹ-CAACAGTATACATATGGT GAGCC-3ʹ). We avoided processing DSL isolated from muskox populations where the muskox lungworm, *Umingmakstrongylus pallikuukensis* Hoberg et al., 1995, is known to co-occur with *V. eleguneniensis* (Hoberg et al., 1995; Kutz et al., 2007, 2012, 2013). These primers and the PCR conditions to follow were previously designed and optimized for *V. eleguneniensis* by collaborators at the United States National Parasite Collection (USNPC; Ingrid Asmundsson, Art Abrams, EPH). PCR reactions followed the same protocol described above for the ITS-2 locus. The amplification conditions used were an initial 2 min denaturation at 94 °C, followed by 38 cycles of 94 °C for 20s, 52.5 °C for 30s, and 68 °C for 40s. A final extension phase of 7 min at 68 °C was followed by cooling to 10 °C.

### 2.4 Sequencing and sequence analysis

DNA templates for direct sequencing of the ITS-2 region were cleaned using ExoSAP-it® or column purified using e. Z.N.A MicroElute® Cycle Pure Kit (Omega Biotek) following the manufacturers’ protocols. Amplicons were sequenced from both ends using the same primers used for PCR amplification for each region with BigDye Terminator Cycle Sequencing (Applied Biosystems). Sequences of complete ITS-2 and partial COI were edited using FinchTV 1.4.0 and MEGA 6.0 (Tamura et al., 2013).

## 3 Results

### 3.1 Caribou

A total of 1485 fecal samples was obtained from 67 populations/herds of Grant's, barren-ground, and woodland caribou distributed from Alaska to Newfoundland, including every Canadian province and territory where caribou are extant, and Greenland (Table 1, S1, S2, S3). These populations covered three of the four caribou ecotypes: migratory tundra, mountain, and boreal caribou (Festa-Bianchet et al., 2011). There is no ecotype designation for Greenland caribou populations. Of the total caribou samples, 354 (23.8%) were positive for protostrongylid DSL (Table 1, S1, S2, S3). These DSL-positive caribou were distributed across 56 herds encompassing the three subspecies and three ecotypes, from Alaska to Newfoundland. Information on DSL prevalence per subspecies and number of infected populations can also be found in Table 1, Table 2, Table 3

**Fig. 1:**
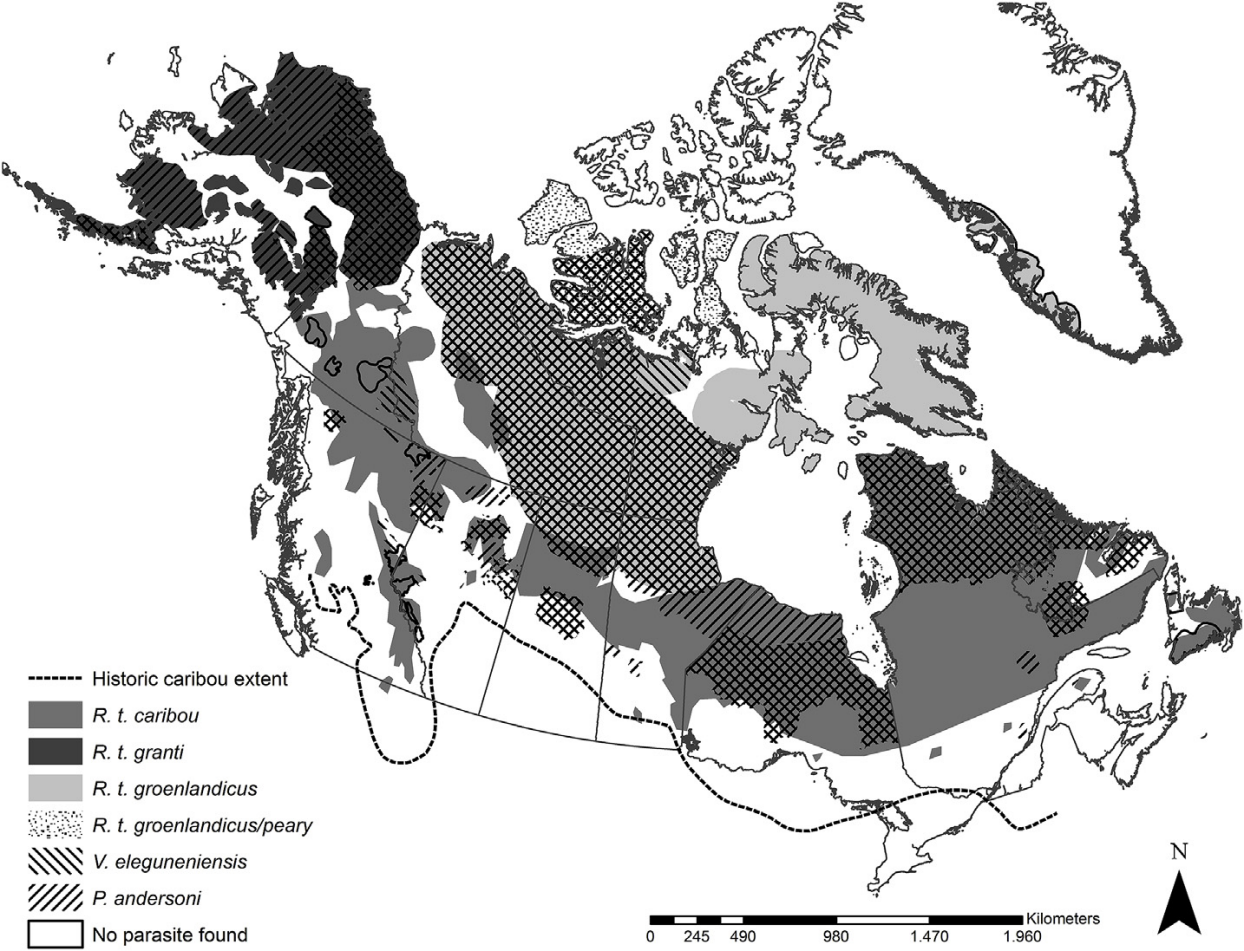
Map of Northern North America, including Alaska, USA, Canada and Greenland depicting the geographic distribution of caribou subspecies (*Rangifer tarandus* sspp.). The distribution of the caribou lungworm, *Varestrongylus eleguneniensis*, and the muscleworm, *Parelaphostrongylus andersoni* in caribou is shown based on compiled data from the present study, and all reports in the literature (Lankester and Hauta, 1989; Lankester and Fong, 1998; Kutz et al., 2007, 2012; 2013; Verocai et al., 2013; Kafle et al., 2017a, Kafle et al., 2017; Turgeon et al., 2018). The historic southern range of distribution of caribou is shown as a dotted line to assist in the discussions on the historical biogeography of *V. eleguneniensis* and *Rangifer*. (See Fig. S1 and Fig. S2 for the distribution maps for *V. eleguneniensis* and *P. andersoni* separately).

**Fig. S1:**
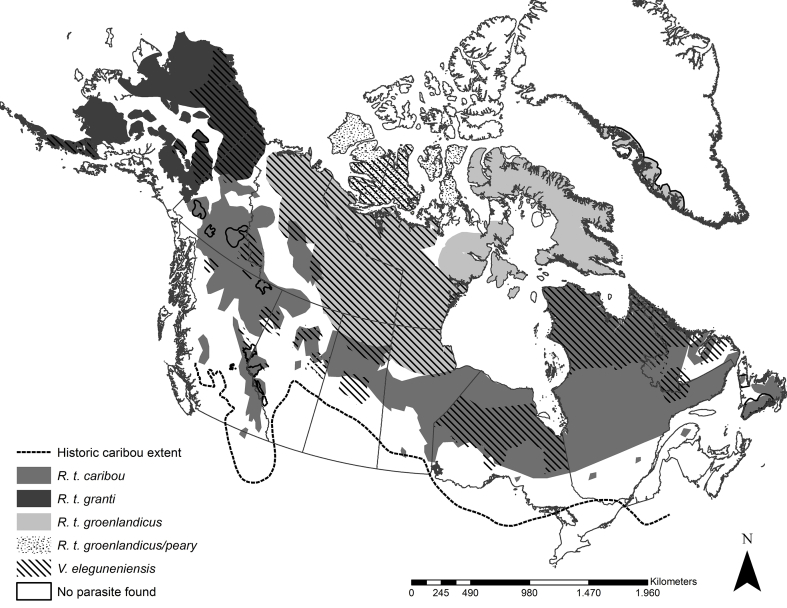
Map of Northern North America, including Alaska, USA, Canada and Greenland depicting the geographic distribution of caribou subspecies (*Rangifer tarandus* sspp.). The distribution of the caribou lungworm, *Varestrongylus eleguneniensis* in caribou is shown based on compiled data from the present study, and all reports in the literature (Kutz et al., 2007, 2012; 2013; ; Kafle et al., 2017a, Kafle et al., 2017; Turgeon et al., 2018). The historic southern range of distribution of caribou is shown as a dotted line to assist in the discussions on the historical biogeography of *V. eleguneniensis* and *Rangifer*.

**Fig. S2:**
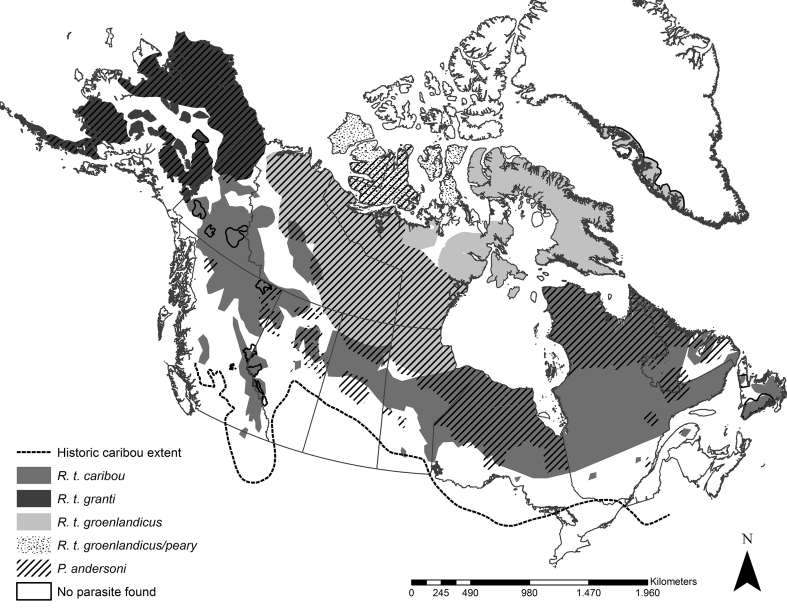
Map of Northern North America, including Alaska, USA, Canada and Greenland depicting the geographic distribution of caribou subspecies (*Rangifer tarandus* sspp.). The distribution of the muscleworm, *Parelaphostrongylus andersoni* in caribou is shown based on compiled data from the present study, and all reports in the literature (Lankester and Hauta, 1989; Lankester and Fong, 1998; Kutz et al., 2007, 2012; Kafle et al., 2017a, Kafle et al., 2017; Turgeon et al., 2018). The historic southern range of distribution of caribou is shown as a dotted line to assist in the discussions on the historical biogeography of protostrongylid lungworms and *Rangifer*.

*Varestrongylus eleguneniensis* was found in caribou populations from Alaska to Quebec/Labrador (Fig. 1, Fig. S1.), including new geographic records for the parasite in caribou herds across North America (Table 1, S1, S2, S3). More specifically, *V*. *eleguneniensis* was found in 51 caribou belonging to 29 herds of the three subspecies and all ecotypes sampled. *Parelaphostrongylus andersoni* was found in 107 caribou, distributed in 36 herds, and also in all subspecies and ecotypes from Alaska to Newfoundland (Fig. 1, Fig. S2.). Co-infections of *V. eleguneniensis* and *P. andersoni* were diagnosed in 6 animals of different populations, including the three subspecies and ecotypes sampled. *Elaphostrongylus rangiferi* Mitskevitch, 1960 was found only on the island of Newfoundland, province of Newfoundland and Labrador, and this is the first report in the Gregory herd (Table S3). Dorsal-spined larvae (12) in four animals of three woodland caribou populations from BC (mountain ecotype) were determined to be *Parelaphostrongylus* sp., but could not be assigned to species due to incomplete or noisy sequences; however ambiguous SNPs were verified at sites relevant for the discrimination between *P. andersoni* and *Parelaphostrongylus odocoilei* (Hobmaier and Hobmaier, 1934); data not shown (Table S3). Additionally, DSL from two woodland caribou populations of Alberta (mountain ecotype) could not be determined because of loss of DSL and PCR and/or sequencing failure.

DSL of both *V. eleguneniensis* and *P. andersoni* were detected in adult females and males, yearlings, and calves across the seasons, regardless of caribou subspecies or ecotype (in a wide variation of larval output, information not provided). Additional data on a few individual caribou is worth mentioning. A DSL-negative caribou recaptured one year later was found infected with *V. eleguneniensis*. Similarly, another DSL-negative female recaptured two years later was then infected by *P. andersoni*, and yet another one was found infected by *P. andersoni* in two captures two years apart.

### 3.2 Muskoxen

A total of 159 fecal samples was acquired from populations of *O. m. moschatus* endemic to the Northwest Territories and Nunavut and *O. m. wardi* endemic to Nunavut, and introduced to Alaska, Northwest Territories, Quebec and western Greenland (Table 2). DSL were found in 104 samples (72.2%) from endemic and introduced populations of the two subspecies. In most regions, DSL prevalence ranged from approximately 70% up to 100% (Table 2). *Varestrongylus eleguneniensis* was confirmed in *O. m. wardi* populations from Quebec and Alaska, with 19 larvae sequenced from 19 different animals. Larval output in muskoxen from these two areas ranged from just over 1 to 355.6 LPG, and moderate to high counts were frequent.

DSL in populations of *O. m. moschatus* from mainland Northwest Territories and Nunavut were not identified because *V. eleguneniensis* has previously been identified in these populations Kutz et al. (2007). These come from areas where *U. pallikuukensis* co-occurs with *V. eleguneniensis* (Central Canadian Arctic) and are reported elsewhere (Kutz et al., 2013). *Protostrongylus stilesi* Dikmans, 1931 was found in an Alaskan muskox population, in co-infections with *V. eleguneniensis*. No DSL were found in any muskox samples from Greenland.

### 3.3 Moose

We tested 264 moose samples from Alaska, Northwest Territories and Alberta, encompassing populations of two of the four subspecies occurring in North America: the Yukon-Alaska moose, *A. a. gigas*, and the western moose, *A. a. andersoni* (Table 3).

Among moose, DSL were found in both subspecies: *A. a. gigas* from Alaska, and *A. a. andersoni* from the Northwest Territories and Alberta. Due to the subspecific diversity and great distances between sampling locations, findings will be treated separately (Table 3).

Regarding the Alaskan samples, 9 out of 245 (3.7%) moose were positive for DSL. All DSL-positive moose were adults. Of these, five DSL had their identity determined, with *V. eleguneniensis* being confirmed from a single adult female (GMU11), and *P. andersoni* confirmed in four animals (a male from GMUs 11, a female from GMU 12, and a female from 20A). Larval counts were lower than 1 LPG and species identity could not be confirmed from four animals either because of larval loss or failure in the molecular processing (from DNA extraction to sequencing).

A single adult male moose from Northwest Territories was infected with DSL (1/17), identified as *P. andersoni.* From the two animals sampled from Alberta, an adult male was positive for *P. andersoni*, and the other animal, a male yearling, was infected with the protostrongyline *Orthostrongylus macrotis* (Dikmans, 1931) Dougherty and Goble, whose larvae do not possess a dorsal spine at the insertion of the tail tip (Carreno and Hoberg, 1999).

### 3.4 DNA sequence results

Representative sequences were deposited on GenBank under accession numbers MN891731- MN891761 for ITS-2 and MN893782- MN893806 for COI of *V. eleguneniensis*, respectively, and MN900346- MN900386 for ITS-2 of *P. andersoni.*

## 4 Discussion

### 4.1 *General findings on* Varestrongylus eleguneniensis *in caribou herds*

We found *V. eleguneniensis* across caribou populations of three different subspecies and ecotypes, corroborating observations based on less extensive sampling regimes (Kutz et al., 2007, 2013; Verocai et al., 2014b). Our findings support the wide geographic range previously demonstrated for *V. eleguneniensis* by these authors and further expand it to areas of northern and interior Alaska, and across the boreal forests of Canada, including the provinces of British Columbia, Saskatchewan, Ontario, and Quebec, and multiple areas of Alberta. As suggested by Kutz et al. (2007) and Verocai et al. (2014b), multiple studies that reported *P. andersoni* or *P. odocoilei* in caribou based on presence of DSL in the feces should be reconsidered. These studies occurred prior to the discovery and recent description of *V. eleguneniensis*, and presumed that, outside of the island of Newfoundland, any DSL in caribou feces was *P. andersoni,* or less likely *P. odocoilei* (Ball et al., 2001; Gray and Samuel, 1986; Jenkins et al., 2005; Johnson et al., 2010; Kutz et al., 2007; Lankester et al., 1976; Lankester and Fong, 1989, 1998; Lankester and Hauta, 1989). Current data for *V. eleguneniensis,* using molecular and more refined morphological examination, indicates considerable spatial overlap with *P. andersoni* (Ball et al., 2001; Gray and Samuel, 1986; Jenkins et al., 2005; Johnson et al., 2010; Kutz et al., 2007; Lankester et al., 1976; Lankester and Fong, 1989, 1998; Lankester and Hauta, 1989). While we further demonstrate the value of molecular diagnostic tools for identification of protostrongylids, recent studies on detailed morphology of DSL when hosts may be co-infected with two species are equally useful tools, and the choice of methods may be related to logistical and financial aspects of a study or health survey (Kafle et al., 2015, 2017a).

Our findings of *V. eleguneniensis* across a vast distribution, encompassing different caribou subspecies and ecotypes, support a continuous distribution across partially overlapping mainland populations of caribou in Canada and the US. This includes the large migratory caribou herds in the Arctic and Subarctic, whose winter range may overlap with range of non-migratory herds, and those who undergo short migrations such as the boreal forest and mountain ecotypes. The largely overlapping distribution of the caribou-*V. eleguneniensis* assemblage may be a product of a concomitant historical geographic colonization by this assemblage after recession of the continental ice, or initial geographic colonization by the host, with subsequent colonization by the parasite, followed by its spread across much of the host's range (Asmundsson et al., 2008; Hoberg et al., 2008, 2012; Kutz et al., 2007, 2012). Further pieces for this mosaic are the independent events of colonization of new hosts – muskoxen and moose in sympatry with geographically distinct caribou populations (Hoberg, 2010; Hoberg et al., 2012; Kutz et al., 2007; Kutz et al., 2013; Verocai et al., 2014b; present study).

### 4.2 *Absence of* Varestrongylus eleguneniensis *in caribou herds*

*Varestrongylus eleguneniensis* was not detected in 38 caribou populations. Given the opportunistic nature of our sampling, despite geographically extensive and often relatively site intensive field collections, the true absence of *V. eleguneniensis* in many of the assessed caribou populations cannot be confirmed. Nevertheless, for some of these herds, various historical and current factors linked to the environment, and the definitive and intermediate host history and ecology, may explain the apparent absence.

Our results provide further evidence for absence of *V. eleguneniensis* in Greenland caribou, corroborating previous conclusions (Kutz et al., 2012). In this case, absence may be a result of either parasite loss during or after colonization, or historical absence, i.e. the founder caribou that colonized the region were never infected with the parasite. With respect to parasite loss, this may have been because of host and/or parasite populations being too low to maintain the life-cycle, absence or low numbers of gastropods, or (episodic or constant) unfavorable environmental conditions. A previous study by Steele et al. (2013) hypothesized that the common abomasal nematode of caribou, *Ostertagia gruehneri* Skrjabin, 1929, was lost during geographic colonization of the region by caribou approximately 4000–7000 years ago, and subsequently recolonized one of the herds with introduction of infected reindeer to the area. True absence of *V. eleguneniensis* is also likely for Peary caribou (not sampled in this study) as previously suggested due to climate and probable absence of gastropods on islands of the High Arctic and Greenland (Kutz et al., 2012). However, the recent emergence of this parasite in muskoxen and caribou on Victoria Island, Nunavut, presumably because of increasingly permissive climatic conditions for parasite development (Hoberg and Brooks, 2015; Kutz et al., 2013), may facilitate the exposure of Peary caribou to this lungworm in the future (Kafle et al., 2017a).

*Varestrongylus eleguneniensis* was not found in Newfoundland caribou; however, these were insufficiently sampled in the present study (n = 11) and need to be more comprehensively assessed. Previous studies on protostrongylid species infecting Newfoundland caribou have relied solely on morphometrics for identification of DSL (Ball et al., 2001; Lankester and Fong, 1998; Lankester and Northcott, 1979), and also were conducted prior to the discovery and description of *V. eleguneniensis* (Kutz et al., 2007; Verocai et al., 2014b). There is considerable overlap in DSL measurement range between *V. eleguneniensis* [281–400 μm, as per (Kutz et al., 2007), and 355–394 μm as per (Kafle et al., 2015)] and *P. andersoni* [308–382 μm, as per (Prestwood, 1972)], and to a lesser extent with *E. rangiferi* [381–490 μm, as per (Lankester and Northcott, 1979) from caribou with identity of adults confirmed]. Therefore, it is possible that DSL of *V. eleguneniensis* were misidentified among these of *P. andersoni* and *E. rangiferi*, and this minute lungworm may in fact be present in Newfoundland caribou. Conversely, a potential absence of *V. eleguneniensis* on this island could be due to historical absence within caribou of the North American lineage (NAL) that first colonized the island, or parasite loss after colonization of the island around 12–20 thousand years ago (Ka) (Yannic et al., 2014).

A recent study suggested that *V. eleguneniensis* may also be absent from the Atlantic-Gaspésie herd of Quebec (Turgeon et al., 2018). DSL were found in 9 of 32 sampled caribou and a subsample of these larvae (5 DSL/caribou for 8 animals, 1 DSL for 1 animal) were identified by molecular techniques as *P. andersoni*. This apparent absence could be explained by either historical absence in founder caribou or loss in modern or recent times. The Gaspésie population, which originates from the NAL caribou lineage, has declined dramatically in modern times and is the only remaining caribou herd south of the Saint Lawrence River (COSEWIC, 2011; Festa-Bianchet et al., 2011). This loss of connectivity with other caribou populations is a result of extirpation events and habitat fragmentation in the last centuries, and may have impacted the persistence of *V. eleguneniensis*, if once present. In contrast, *P. andersoni* seems to have persisted in this herd, which is parapatric with white-tailed deer populations that could have assisted in sustaining this muscle-worm.

Despite a reasonable sample size (n = 122), *V. eleguneniensis* was not found in any of the five sampled Manitoban herds, including three of the boreal forest ecotype and two of the migratory tundra ecotype. However, the parasite is common in other allopatric and parapatric woodland caribou populations, including populations in the neighboring provinces of Ontario and Saskatchewan (Festa-Bianchet et al., 2011). One of the Manitoba caribou populations, Norway House, had the highest prevalence of DSL seen among woodland caribou populations (70%), but these all sequenced as *P. andersoni*. This high DSL prevalence may have masked the presence of *V. eleguneniensis* in this herd, as we only sequenced larvae (n = 17) from five out of 21 DSL-shedding caribou. Regardless of the apparent absence of *V. eleguneniensis* in the sampled woodland caribou populations in Manitoba, the parasite is present in northern areas of that province, as it occurs in both the Beverly and Qamanirjuaq barren-ground herds (Kutz et al., 2007, present study). The winter range of the Qamanirjuaq herd partially overlaps with the Cape Churchill woodland caribou, whose range partially overlaps with the Pen Island herd. Therefore, there is potential for the presence of *V. eleguneniensis* in these herds, despite it not being detected in the current study.

### 4.3 Muskoxen

*Varestrongylus eleguneniensis* was commonly found in muskox populations, corroborating previous knowledge (Kutz et al., 2007, 2012, 2013; Verocai et al., 2014b). It is probable that *V. eleguneniensis* originated in caribou and subsequently colonized muskoxen on a variety of different time scales. In the central Canadian Arctic, endemic *O. m. moschatus* have co-existed with caribou for millennia, and, therefore, this host colonization event has historical roots and may be prior to or coincidental with the recolonization of these areas by the two species after recession of the Cordilleran and Laurentide continental ice sheets in the late Pleistocene and early Holocene. In this area, muskoxen are also infected with the cyst-forming protostrongylid lungworm *U. pallikuukensis*, a relictual species with an ancient association with muskoxen (Hoberg et al., 1995, 2008; Kutz et al., 2005, 2007). The recent colonization events by *V. eleguneniensis* of translocated muskox populations were briefly discussed by Verocai et al. (2014b). The presence of *V. eleguneniensis* in Nunavik muskoxen (introduced from Ellesmere Island) was previously reported by Kutz et al. (2007) and Verocai et al., 2014b, Verocai et al., 2014a, however, for the first time the lungworm is confirmed in the two sympatric caribou herds (Rivière-aux-Feuilles and George River), suggesting their potential role as source for muskox infection. Similarly, the presence of *V. eleguneniensis* in muskox populations in Alaska (originally introduced from Greenland) may be explained by initial colonization events from sympatric caribou herds. We detected *V. eleguneniensis* in the Teshekpuk caribou herd but not the Western Arctic caribou herd. However, because of the presence in sympatric muskoxen, we expect that *V. eleguneniensis* is present in the latter. In Alaska, natural populations of *O. m. moschatus* existed until complete extirpation in the late 1800s (Paul, 2009). It is likely that these muskoxen were infected by *V. eleguneniensis*, similar to their relatives in the Central Canadian Arctic. Based on this knowledge, we can predict future events of colonization in natural and translocated muskox populations of both subspecies, in areas of sympatry with caribou.

### 4.4 Moose

The study confirms that *V. eleguneniensis* is rare in moose, and that this parasite may be a spill-over from sympatric caribou and not likely to persist in moose in the absence of caribou, as previously suggested (Kutz et al., 2007). The only moose found infected by *V. eleguneniensis* was also from Alaska, likely sympatric with caribou herds from Interior Alaska (e.g. Chisana, Nelchina or Mentasta). However, only *P. andersoni* has been found in two of these caribou herds (Kutz et al., 2007; present study), while the remaining herd has not been assessed for the presence of protostrongylids. Despite being an isolated finding, the presence of *V. eleguneniensis* in moose from this region indicates that this parasite is likely present in sympatric caribou herds, which act as source for infection of moose.

For the first time, *P. andersoni* infections were confirmed in two moose subspecies, the Yukon-Alaska moose (*A. a. alces*) and the western moose (*A. a. andersoni*) from the Northwest Territories and Alberta (AB). All of these moose are sympatric with either barren-ground or woodland caribou populations, some known to be infected by *P. andersoni* (Kutz et al., 2007; present study), indicating their potential source for moose. Previously, the only unconfirmed reports of *P. andersoni* in moose came from the eastern moose (*A. a. americanus*) in Newfoundland (Lankester and Fong, 1998). In addition, we found *O. macrotis* in a yearling moose from Peace River, AB, which is likely the northernmost record for this species (Samuel et al., 1976).

### 4.5 *Parelaphostrongylus andersoni* in caribou herds and other protostrongylids

The widespread findings of *P. andersoni* across caribou range corroborate previous assumptions of an extensive geographic distribution across northern North America (Asmundsson et al., 2008; Hoberg et al., 2008; Kutz et al., 2007; Lankester, 2001). Since the first discovery of *P. andersoni* in caribou from Ontario and Quebec by Lankester and Hauta (1989), knowledge of the distribution of this host-parasite assemblage has increased dramatically (Kutz et al., 2007; Lankester and Fong, 1989). Our contributions provide a finer-scale picture of its presence across caribou range, including various new herd and geographic records for this parasite. For most of the caribou range, *P. andersoni* and *V. eleguneniensis* co-occurred, however, *P. andersoni* was more commonly confirmed from DSL-infected caribou, and possibly due to sampling bias, it was present in some herds where *V. eleguneniensis* was not sequenced, as previously reported from surveys in barren ground, Grant's and woodland caribou populations (Bondo et al., 2019; Kutz et al., 2007; Turgeon et al., 2018). However, the opportunistic sampling strategies of those studies do not allow for a powerful comparison of distribution or prevalence of these two protostrongylids. In addition, even though our sampling strategy was not designed specifically to diagnose co-infections, *P. andersoni* and *V. eleguneniensis* co-infections were determined in caribou of different subspecies and ecotypes. To date, there has been no evidence whether or not dual infections by these protostrongylids have additive or synergistic impacts on caribou health, but this hypothesis was postulated by Kutz et al. (2012).

Despite the fact that muskoxen are sympatric with infected caribou populations across much of their range, absence of *P. andersoni* in muskoxen is well supported by the present results and corroborates the literature (Hoberg et al., 1995, 2002; Kutz et al., 2007, 2012, 2013). To date, *P. andersoni* has never been reported from any caprine hosts (Kutz et al., 2012; Lankester, 2001), but its close relative, *P. odocoilei,* is found in Dall's sheep (*Ovis dalli* Nelson, 1884) and bighorn sheep (*Ovis canadensis* Shaw, 1804). The range of muskoxen and *P. odocoilei* do not overlap (Jenkins et al., 2005, 2006; Kutz et al., 2001, 2004). However, as, Dall's sheep in its northern range and sympatric muskoxen share *Pr. stilesi* (Hoberg et al., 2002; Jenkins et al., 2006; Kutz et al., 2001, 2012).

*Parelaphostrongylus odocoilei* was not confirmed in caribou samples in this study. However, larvae of *Parelaphostrongylus* of undetermined species were found in woodland caribou of three British Columbia populations. The presence of *P. odocoilei* in other populations cannot be ruled out without further sequencing. The only two unequivocal reports of this parasite in caribou seem to be incidental cases in areas of sympatry with *Odocoileus* and wild caprines in western Canada (Gray and Samuel, 1986; Jenkins et al., 2005).

### 4.6 Insights on the biology and epidemiology of protostrongylids in caribou

Although not designed specifically to address the ecology of protostrongylids in their ungulate hosts, our results provide some insights on the biology and epidemiology *V. eleguneniensis* and *P. andersoni*. There is strong evidence that DSL of both parasites are shed by caribou of all age classes, sexes, and during all seasons. Previous studies on *P. andersoni* in caribou based on the experimental infection of a single calf suggested a short patency, with a sharp decline in larval output about a month after reaching patency (Lankester and Hauta, 1989), but longer patency was achieved in infected white-tailed deer (i.e., more than one year) (Nettles and Prestwood, 1976; Pybus and Samuel, 1981). Studies on experimental infection of captive wild ungulates with protostrongylids are challenging and logistically difficult, and often include only a few individuals. Therefore, although informative, their results should be cautiously interpreted as these likely do not simulate natural infection conditions (e.g., infective dose, constant exposure to infection), and often do not investigate the life-cycle in different suitable sympatric host species. Moreover, Ball et al. (2001) in attempting to determine caribou age by fecal pellet size suggested that *P. andersoni* was shed only by calves and yearling caribou in Newfoundland, a paradigm that is contradicted by our findings of this parasite in adult caribou and moose.

Prevalence of *V. eleguneniensis* and larval output in muskox populations are typically much higher than in sympatric caribou, suggesting that the parasite could persist in muskoxen in the absence of caribou. This may be further supported for the longer patency and larval output of *V. eleguneniensis* in an experimentally infected muskox in comparison to reindeer (Kafle et al., 2017). Findings of *V. eleguneniensis* and *P. andersoni* in moose were scarce and associated with very low larval output. Therefore, it is unlikely that moose contributes to the transmission and maintenance of either species (low prevalence, low LPG), and could not support parasite persistence in the absence of alternate sympatric hosts (e.g., caribou/muskoxen for *V. eleguneniensis* or caribou/*Odocoileus* spp. for *P. andersoni*).

*Biogeography of* Varestrongylus eleguneniensis - *linking the past, present and future*.

Establishing the current biogeography and the host associations of *V. eleguneniensis*, and indirectly of *P. andersoni*, allow us to make inferences about the past. The distribution of any parasitic species is a reflection the distribution of its host(s), but current host ranges have been shaped by complex processes across historical times until the present (Verocai et al., 2018a, b). Therefore, the study of the distribution of such host-parasite assemblages must take into consideration the deep past, the recent past and the numerous variables that are currently affecting these systems (Hoberg et al., 2012, 2015, 2017; Hoberg and Brooks, 2008, 2010, 2013; Kutz et al., 2009, 2012, 2014).

Understanding the complex history and biogeography of *V. eleguneniensis* is fundamental to critically assess its presence and potential absence in caribou subspecies (e.g., *Rangifer tarandus pearyi* (Allen, 1902)), supported by recent findings by Kafle et al. (2017a), or other specific caribou populations. A historically deep association of *V. eleguneniensis* and *Rangifer* has been supported by the phylogenetic inference for *Varestrongylus*, and by the limited molecular phylogenetic data (Verocai et al., 2014a, 2014b, 2018b). Likely, this association existed prior to the multiple waves of expansion of this ungulate into the Nearctic across a wide window of 2–3 million years during the Pleistocene (Banfield, 1961; Flagstad and Røed, 2003; Weckworth et al., 2012). Therefore, *V. eleguneniensis* may be, in fact, a Beringian endemic associated with the genus *Rangifer* (Hoberg et al., 2012; Kurtén and Anderson, 1980; Weckworth et al., 2012; Yannic et al., 2014). However, the biogeographic history of caribou is also very complex and the species survived the Glacial Maxima in multiple refugia, north and south of the continental ice-sheets, as did many other components of the current Nearctic fauna (Banfield, 1961; Hoberg et al., 2012; Klütsch et al., 2012, 2016; Polfus et al., 2016; Shafer et al., 2010; Yannic et al., 2014).

Sampling across the vast *V. eleguneniensis* range has generated material to further investigate the three hypotheses postulated by Verocai et al. (2014a), in which population genetics of the parasite may support one of the three hypotheses: i) the parasite distribution was restricted to Beringia with caribou of the Beringian-Eurasian lineage, and expanded eastwards and southwards across the continent after deglaciation just over 10 thousand years ago, ii) the parasite distribution was restricted to the south of the continental ice in caribou of the North American lineage and later expanded northwards, or iii) the parasite was present in caribou populations north and south of the ice sheets and expanded in all directions until covering its current range. Further, satellite-hypotheses of multiple refugia within refugia may arise after a powerful study on the genetic diversity and signatures of *V. eleguneniensis* populations, and may be an indicator of caribou population genetics and phylogeography. Nevertheless, as highlighted by Verocai et al. (2014b), there is still a need for a broader assessment for the presence of *V. eleguneniensis* in Eurasian reindeer, which ranges from Fennoscandia to Eastern Russia. If *V. eleguneniensis* is present across most of *Rangifer* range, novel hypotheses for its historical biogeography will have to be articulated and tested.

Overall, the recovery and sustainability of caribou populations across the continent is uncertain (Festa-Bianchet et al., 2011; Gustine et al., 2014; Hervieux et al., 2013; Vors and Boyce, 2009), raising serious concerns about the species conservation and long-term persistence. Currently, caribou's northern range is facing unprecedented, fast, and ongoing changes that may also impact the geographic distribution and host-associations for *V. eleguneniensis*. In fact, it has been recently demonstrated that *V. eleguneniensis* is undergoing a northward range expansion, as climatic conditions have become permissive to its establishment on an Arctic island (Hoberg and Brooks, 2015; Kutz et al., 2013). Moreover, the anthropogenic introduction of muskoxen into multiple areas within the range of the caribou-*V. eleguneniensis* assemblage may impact the population dynamics of this lungworm species, as another suitable host is contributing to the environmental contamination with larvae of the parasite. Therefore, an increased infection pressure by *V. eleguneniensis* may be expected in these two-host areas (Kutz et al., 2007, 2012, 2013; Verocai et al., 2014b).

Across their southern range, woodland caribou have suffered considerable geographic retraction that began during European colonization and has continued to present (Banfield, 1961; COSEWIC, 2011; Festa-Bianchet et al., 2011). Concurrently, where *V. eleguneniensis* is thought to have the caribou as its only epidemiologically relevant definitive host (i.e., considering moose infections incidental), direct and indirect anthropogenic pressures including exploitation of renewable (logging, eco-tourism) and non-renewable natural resources (mining, and oil and gas industries) continue to profoundly impact caribou, including an event of local extinction and unlikely long-term persistence of multiple herds (Canada, 2017; COSEWIC, 2011; Festa-Bianchet et al., 2011; Hebblewhite et al., 2010; Hervieux et al., 2013). We postulate that with the ongoing range retraction of caribou, the distribution of *V. eleguneniensis* will also continuously retract northwards alongside its primary host.

Combining the current knowledge and predictions on host populations, the geographic range of *V. eleguneniensis* is gradually shifting northwards. At the southern limit of its range, where woodland caribou are considered to be the only suitable host, *V. eleguneniensis* is expected to parallel the range retraction of caribou. At the northern limit of its range the lungworm persists in a multi-host caribou/muskox system, and this, together with increasingly permissive climatic conditions, is likely to facilitate further northward expansion (Kutz et al., 2013; Kafle, Kutz, Leclerc, unpublished data). We anticipate a continuous northward shifting range for *V. eleguneniensis* into the next decades and centuries. The long-term persistence of this lungworm may ultimately only be possible in its northernmost range. This northern expansion and potential persistence of *V. eleguneniensis*, is supported by empirical data, predictions, and quantitative modelling for protostrongylid species under changing climatic conditions (Hoberg and Brooks, 2013, 2015; Kutz et al., 2005, 2009, 2014; Molnár et al., 2013). Complex host-parasite distributions represent the interaction of capacity by parasites to utilize resources present in a spectrum of potential hosts (defined by ecological fitting) and opportunity, which for *V. eleguneniensis*, represents the combination of shifting distributions of permissive environments and development of new interfaces for hosts and parasites across landscapes (see discussions of the Stockholm Paradigm in Araújo et al., 2015; Brooks et al., 2014). Ecological perturbation, range expansion and host colonization are fundamental to the process of faunal assembly and the outcomes for this northern host-parasite fauna are documented and anticipated under a regime of accelerating climate forcing (Araújo et al., 2015; Hoberg and Brooks, 2015; Hoberg et al., 2017).

The caribou lungworm, *V. eleguneniensis* is widely distributed across caribou range in North America. We have expanded the knowledge of its biogeography substantially, and provided a strong baseline for monitoring geographic distribution and predicting future biogeographic scenarios under accelerating change at high latitudes of North America. The biogeography of *V. eleguneniensis* is a result of an intricate historical association with caribou, independent events of colonization of alternate hosts, and ongoing climatic and anthropogenic perturbations. Together, these will likely continue to influence the dynamic biogeography of this lungworm species, which may be a powerful model for studying impacts of climate and people on complex faunal assemblages.

## Funding

This research is part of G. Verocai's PhD Thesis, and was supported by the Faculty of Veterinary Medicine of the University of Calgary, Alberta Innovates Health Solutions, Alberta Conservation Association – Grants in Biodiversity and the W. Garfield Weston Fellowship for Northern Conservation/Wildlife Conservation Society Canada, and the CircumArctic Rangifer Monitoring and Assessment Network (CARMA, www.carmanetwork.com), NSERC Canada International Polar Year Funding, and partially funded by Alberta Innovates and NSERC Discovery Grant and NSERC Northern Supplement secured by S.J. Kutz; the Beringian Coevolution Project (DEB- Biotic Surveys and Inventory- 0415668) with funding from the National Science Foundation to J. A. Cook (University of New Mexico) and E. P. Hoberg (USNPC). Our study was completed through the Integrated Inventory of Biomes of the Arctic (NSF, DEB-Biodiversity Discovery and Analysis – 1258010) to J. A. Cook, E. P. Hoberg, K. E. Galbreath (Northern Michigan University) and E. Dechaine (Western Washington University).

## Declaration of competing interest

The authors declare no conflict of interest.
