# Supplementary material for: The biogeography of the caribou lungworm, Varestrongylus eleguneniensis (Nematoda: Protostrongylidae) across northern North America
Source: Int J Parasitol Parasites Wildl. 2020 Jan 8;11:93–102. doi: 10.1016/j.ijppaw.2020.01.001 (PMC6965202; doi:10.1016/j.ijppaw.2020.01.001)
Supplement: Multimedia component 1 [file mmc1.docx]

Table S1. Grant’s caribou (*Rangifer tarandus granti*) fecal samples included in the study: information on subspecies and origin, and Baermann results (prevalence of dorsal-spined larvae; DSL). Molecular identification of DSL was based on sequences of the ITS-2 region of the nuclear ribosomal DNA

| ***R. t. granti*** | **Ecotype** | **Geographic Range** | **Month, Year** | **N** | **DSL (%)** | ***V. ele.***  **(DSL/host)** | ***P. and.***  **(DSL/host)** |
| --- | --- | --- | --- | --- | --- | --- | --- |
| Western Arctic | Migratory Tundra | AK | Sept, 2007 | 11 | 1 (9) | - | 6; 2 |
|  |  |  | Sept, 2010 | 9 | 2 (22.2) | - | 4; 1 |
|  |  |  | June, 2011 | 1 | 0 | - | - |
| Teshekpuk | Migratory Tundra | AK | June, 2010 | 39 | 10 (25.6) | - | 13; 6 |
|  |  |  | June, 2011 | 21 | 9 (42.9) | 1; 1* | 9; 5* |
| Central Arctic | Migratory Tundra | AK | June, 2010 | 14 | 2 (14.3) | 1; 1 | 6; 1 |
|  |  |  | April, 2011 | 16 | 9 (56.3) | - | 14; 7 |
| Porcupine | Migratory Tundra | AK-YT-NT | Sept, 2008 | 13 | 1 (7.7) | 2; 1 | - |
|  |  |  | Sept, 2009 | 10 | 1 (10) | - | 1; 1 |
| Fortymile | Mountain | AK-YT | May, 2011 | 8 | 6 | 2; 1 | 8; 4 |
|  |  |  | Oct, 2011 | 7 | 1 (14.3) | - | - |
|  |  |  | May, 2012 | 14 | 1 (7.1) | - | - |
| Nelchina | Mountain | AK | April, 2011 | 19 | 3 (15.8) | - | 7; 3 |
|  |  |  | Oct, 2011 | 5 | 1 (20) | 1;1* | 1;1* |
| Delta | Mountain | AK | April, 2011 | 13 | 7 (53.8) | 6; 3 | 5; 2 |
| Denali | Mountain | AK | March, 2011 | 3 | 1 (33.3) | - | 3; 1 |
| White Mountain | Mountain | AK | April, 2011 | 1 | 0 | - | - |
| **TOTAL**  ***R. t. granti*** |  |  |  | **203** | **54 (26.6)** | **13; 8** | **77; 32** |

*: Co-infections of *V. eleguneniensis* and *P. andersoni*; AK = Alaska, YT = Yukon Territory, NT= Northwest Territories. *V. ele*. = *Varestrongylus eleguneniensis*, *P. and.* = *Parelaphostrongylus andersoni*.

Table S2. Barren-ground caribou (*Rangifer tarandus groenlandicus*) fecal samples included in the study: information on subspecies and origin, and Baermann results (prevalence of dorsal-spined larvae; DSL). Molecular identification of DSL was based on sequences of the ITS-2 region of the nuclear ribosomal DNA

| ***R. t. groenlandicus*** | **Ecotype** | **Geographic Range** | **Month, Year** | **N** | **DSL (%)** | ***V. ele.***  **(DSL/host)** | ***P. and.***  **(DSL/host)** |
| --- | --- | --- | --- | --- | --- | --- | --- |
| Bluenose West, Tuktoyaktuk Peninsula  and Cape Bathurst | Migratory Tundra | NT | May-July, 2009 | 112 | 35 (31.3) | 2; 2 | 4; 2 |
| Bathurst | Migratory Tundra | NT-NU | Aug-Sept, 2008 | 33 | 11 (33.3) | - | - |
|  |  |  | Sept, 2008 | 26 | 1 (4) | - | - |
|  |  |  | Spring, 2009 | 34 | 4 (11.8) | 2; 1 | 1; 1 |
| Bluenose East | Migratory Tundra | NT-NU | Feb, 2009 | 18 | 8 (17.8) | - | - |
| Ahiak | Migratory Tundra | NU | March, 2009 | 36 | 21 (58.3) | 2; 2 | - |
| Beverly-Qamanirjuaq | Migratory Tundra | NT-NU-MB | March, 2009 | 23 | 15 (65.2) | 3; 2 | - |
| Akia-Maniitsoq | NA | Greenland | March-April, 2008 | 47 | 0 (0) | - | - |
| Kangerlussuaq-Sisimiut | NA | Greenland | March, 2009 | 50 | 0 (0) | - | - |
| **TOTAL**  ***R. t. groenlandicus*** |  |  |  | **375** | **95 (25.3)** | **9; 7** | **5; 3** |

NT= Northwest Territories, NU = Nunavut, MB = Manitoba, NA = not applicable, *V. ele*. = *Varestrongylus eleguneniensis*, *P. and.* = *Parelaphostrongylus andersoni*.

Table S3. Woodland caribou (*Rangifer tarandus caribou*) fecal samples included in the study: information on subspecies and origin, and Baermann results (prevalence of dorsal-spined larvae; DSL). Molecular identification of DSL was based on sequences of the ITS-2 region of the nuclear ribosomal DNA.

| ***R. t. caribou*** | **Ecotype** | **Geographic Range** | **Month, Year** | | **N** | **DSL (%)** | ***V. ele.***  **(DSL/host)** | ***P. and.***  **(DSL/host)** |
| --- | --- | --- | --- | --- | --- | --- | --- | --- |
| South Nahanni | Mountain | YT/NT | Oct, 2008 | | 17 | 2 (12) | 1; 1 | - |
| Aishihik | Mountain | YT | Oct, 2011 | | 4 | 0 (0) | - | - |
| Laberge | Mountain | YT | Oct, 2011 | | 4 | 0 (0) | - | - |
| Finlayson | Mountain | YT | Oct, 2011 | | 2 | 0 (0) | - | - |
| Coal River | Mountain | YT/NT | Oct, 2011 | | 2 | 0 (0) | - | - |
|  |  |  | Nov, 2012 | | 1 | 1 (100) | 1; 1 |  |
| Level-Kawdy | Mountain | BC | Oct, 2011 | | 20 | 1 (5) | - | 1; 1 |
|  |  |  | Feb-March, 2012 | | 23 | 1 (4.3) | 5; 1 | - |
|  |  |  | March, 2012 | | 5 | 0 (0) | - | - |
|  |  |  | April, 2012 | | 4 | 0 | - | - |
| Liard Plateau | Mountain | YT-BC | Dec, 2010 | | 15 | 1 (6.7) | 1; 1 | - |
| Telkwa | Mountain | BC |  | | 5 | 0 | - | - |
|  |  |  |  | | 5 | 1 (20) *^P^* | - | - |
| Kennedy Siding | Mountain | BC |  | | 8 | 1 (12.5) *^P^* | - | - |
| Moberly | Mountain | BC | March, 2011 | | 5 | 0 | - | - |
|  |  |  | March, 2012 | | 1 | 1 (100) | 1; 1 | - |
| Quintette | Mountain | BC | March, 2010 | | 3 | 1 (33) | 1; 1 | - |
|  |  |  | March, 2011 | | 1 | 0 | - | - |
| Narraway | Mountain | BC-AB | Jan, 2009 | | 1 | 0 | - | - |
|  |  |  | Dec, 2009 | | 2 | 0 | - | - |
|  |  |  | March, 2010 | | 3 | 0 | - | - |
| A La Peche | Mountain | AB | March, 2010 | | 5 | 1 (20) | - | - |
| North Jasper | Mountain | AB | July, 2009 | | 28 | 2 (7.1) | - | - |
|  |  |  | June, 2011 | | 12 | 0 | - | - |
| North Banff | Mountain | AB | March, 2009 | | 2 | 0 | - | - |
| Bakerville | Mountain | BC | March, 2011 | | 18 | 0 | - | - |
| Purcells South | Mountain | BC | April, 2012 | | 1 | 1(100)*^P^* | - | - |
| Calendar | Boreal Forest | BC | Jan-Feb, 2008 | | 16 | 4 (25) | - | 6; 2 |
|  |  |  | March, 2010 | | 1 | 0 (0) | - | - |
| Maxhamish | Boreal Forest | BC | March, 2010 | | 3 | 0 (0) | - | - |
| Snake-Sahtaneh | Boreal Forest | BC | April, 2008 | | 1 | 0 (0) | - | - |
|  |  |  | Nov, 2008 | | 6 | 2 (33.3) | - | 8; 2 |
|  |  |  | March, 2009 | | 1 | 1 (100) | - | 1; 1 |
|  |  |  | March, 2010 | | 4 | 1 (25) | - | - |
| Chinchaga | Boreal Forest | BC-AB | Feb, 2003 | | 8 | 5 (62.5) | 2; 1* | 5 ,3* |
|  |  |  | March, 2010 | | 3 | 1 (33.3) | - | 2; 1 |
|  |  |  | Feb, 2012 | | 3 | 1 (33.3) | - | 4; 1 |
| Little Smoky | Boreal Forest | AB | Nov, 2010 | | 1 | 1 (100) | 3; 1 | - |
| Slave Lake | Boreal Forest | AB | Feb, 2003 | | 2 | 1 (50) | - | 2; 1 |
| Caribou Mountain | Boreal Forest | AB | Feb, 2001 | | 9 | 2 (22.2) | - | 4; 1 |
| Red Earth | Boreal Forest | AB | Feb, 2000 | | 13 | 2 (15.4) | 3; 1* | 1; 1* |
|  |  |  | Feb, 2004 | | 8 | 2 (25) | - | - |
| WSAR (not specified) | Boreal Forest | AB | Feb, 2003 | | 7 | 0(0) | - | - |
| WSAR - Wabasca | Boreal Forest | AB | Feb, 2000 | | 11 | 2 (18.2) | - | 3; 1 |
| ESAR (not specified) | Boreal Forest | AB | Feb, 2001 | | 13 | 1 (7.7) | - | - |
|  |  |  |  | | 5 | 1 (20) | - | 1; 1 |
| ESAR - Algar | Boreal Forest | AB | Feb-March, 2009 | | 50 | 10 (20) | 12; 6 | - |
| ESAR – Egg Pony | Boreal Forest | AB | Feb-March, 2009 | | 48 | 17 (35.4) | 5; 3* | 7; 5* |
| Cold Lake | Boreal Forest | AB-SK | Feb, 2001 | | 9 | 3 (33.3) | 2; 1 | 2;1 |
|  |  |  | Feb, 2003 | | 5 | 1 (20) | - | 2; 1 |
|  |  |  | Feb, 2004 | | 4 | 0 (0) | - | - |
|  |  |  | Nov, 2010 | | 1 | 1 (100) | 2; 1 | - |
| Smoothstone-Wapeweka | Boreal Forest | SK | Feb, 2013 | | 30 | 14 (46.7) | 6; 2 | 18; 4 |
| The Bog | Boreal Forest | MB | Feb, 2007 | | 20 | 6 (30) | - | 11; 6 |
| Northern Interlake | Boreal Forest | MB | Jan, 2009 | | 30 | 0 (0) | - | - |
| Norway House | Boreal Forest | MB | Jan, 2012 | | 30 | 21 (70) | - | 17; 5 **^§^** |
| Cape Churchill | Migratory Tundra | MB | March, 2010 | | 22 | 9 (40.9) | - | 24; 5 |
| Pen Island | Migratory Tundra | MB-ON | March, 2012 | | 20 | 7 (28.6) | - | 22; 6 |
| Auden | Boreal Forest | ON | April, 2010 | | 41 | 9 (22) | 5; 3 | 10; 4 |
|  |  |  | April, 2012 | | 9 | 3 (33.3) | - | - |
| Pickle Lake | Boreal Forest | ON | April, 2010 | | 38 | 5 (13.7) | 2; 1 | 8; 3 |
|  |  |  | April, 2012 | | 7 | 0 (0) | - | - |
| Cochrane | Boreal Forest | ON | April, 2010 | | 31 | 7 (22.6) | 6; 5 | 5; 2 |
|  |  |  | April, 2012 | | 6 | 0 (0) | - | - |
| Manicouagan | Boreal Forest | QC | March, 2009 | | 3 | 1 (33.3) | - | 2; 1 |
|  |  |  | April, 2011 | | 3 | 0 (0) | - | - |
| Lac Joseph | Boreal Forest | QC | March, 1998 | | 7 | 2 (28.6) | - | 1; 1 |
| Romaine | Boreal Forest | QC | Feb, 2011 | | 7 | 0 (0) | - | - |
|  |  |  | March, 2011 | | 8 | 3 (35.7) | 2; 1 | 3; 2 |
| Saguenay South | Boreal Forest | QC | March, 2011 | | 5 | 2 (40) | - | 3; 2 |
| Leaf River | Migratory Tundra | QC | Oct, 2008 | | 1 | 0 (0) | - | - |
|  |  |  | Winter 2009 | | 31 | 8 (25.8) | - | - |
|  |  |  | Spring 2010 | | 15 | 3 (20) | - | - |
|  |  |  | Summer 2010 | | 30 | 6 (20) | 5; 2 | 8; 3 |
|  |  |  | May, 2011 | | 10 | 3 (33.3) | - | 7; 4 |
| George River | Migratory Tundra | QC-NL | Winter 2009 | | 29 | 2 (6.9) | - | - |
|  |  |  | Spring 2010 | | 15 | 8 (53) | - | - |
|  |  |  | Summer 2010 | | 30 | 11(36.7) | 4; 2 | 5; 2 |
| Grey River | Boreal Forest | NL | Jan, 2009 | | 1 | 1 (100)*^E^* | - | - |
| Lapoile | Boreal Forest | NL | Jan, 2009 | | 3 | 0 | - | - |
| Buchans | Boreal Forest | NL | April, 2009 | | 2 | 1 (50) *^E^* | - | - |
| Topsails | Boreal Forest | NL | March 2010 | | 2 | 1 (50) *^E^* | - | - |
| Gregory | Boreal Forest | NL | March 2010 | | 2 | 1 (50) *^E^* | - | - |
| Gros Morne | Boreal Forest | NL | Feb, 2011 | | 1 | 0 | - | - |
| **TOTAL**  ***R. t. caribou*** |  |  |  | **907** | | **212 (23.4)** | **69; 36** | **193; 72** |

*: Co-infections of *V. eleguneniensis* and *P. andersoni*; *E:* DSL identified as *Elaphostrongylus rangiferi*, *P:* DSL identified as *Parelaphostrongylus* sp., but species not determined. YT = Yukon Territory, NT= Northwest Territories, BC= British Columbia, AB= Alberta, SK= Saskatchewan, MB= Manitoba, ON= Ontario, QC= Quebec, NL= Newfoundland Island, Newfoundland and Labrador. *V. ele*. = *Varestrongylus eleguneniensis*, *P. and.* = *Parelaphostrongylus andersoni*, WSAR = West Side Athabasca River, ESAR: East Side Athabasca River.
